# Commentary: An impressive state‐of‐the‐science account and an exciting springboard for new paths: the present and future of research on early conduct problems – a commentary on Hyde et al. (2025)

**DOI:** 10.1111/jcpp.70123

**Published:** 2026-01-25

**Authors:** Grazyna Kochanska

**Affiliations:** ^1^ Department of Psychological and Brain Sciences The University of Iowa Iowa City IA USA

**Keywords:** Conduct problems, parenting, developmental psychopathology, attachment, internal working models

## Abstract

In this commentary on ‘Annual Research Review: Early conduct problems – precursors, outcomes, and etiology’ by Hyde and colleagues, I discuss the strengths of that review and its heuristic value in inspiring future research directions. The review is an impressive, comprehensive, scholarly, and up‐to‐date broad summary of the current state of developmental science related to conduct problems. By embracing biopsychosocial/ecological perspective and reviewing constructs and processes across multiple levels, it has a cutting‐edge quality and scope. But perhaps even more importantly, it is heuristically fertile: It inspires new compelling questions and can potentially forge new bridges with other perspectives and areas of research. I discuss two such new directions that can complement – without contradicting – the authors' ideas. One, I argue for expanding the focus to relational experiences and parents' and children's emerging representations in the first years of life to elucidate very early origins of maladaptive cascades leading to conduct problems. And two, I suggest complementing the current emphasis on adverse developmental factors – a natural focus in the study of conduct problems – with research on positive socialization forces that can act as powerful buffers against risks for antisocial behavior.

Children's conduct problems pose very significant challenges and entail substantial costs to individuals, families, and societies. Scholars have long sought to explain their causes and developmental mechanisms, with the ultimate goal of informing prevention and treatment. However, the origins of conduct problems are highly complex, and their full understanding has long been a holy grail for scholars pursuing basic research in developmental psychology and psychopathology, as well as for those seeking to translate that knowledge into evidence‐based effective interventions.

Research on developmental trajectories of conduct problems and their treatment is massive; consequently, producing a comprehensive review is a formidable goal. The article ‘Annual Research Review: Early conduct problems – precursors, outcomes, and etiology’ by Hyde and colleagues (Hyde, Trentacosta, & Bezek, 2025) accomplishes this goal admirably. It is a substantial, scholarly, and up‐to‐date contribution to the field, presenting a broad review of developmental science related to conduct problems. It is an excellent resource for research and for graduate training, and an intellectual treat to read.

The review is impressive in its richness, comprehensiveness, cutting‐edge quality, and scope. It embraces a biopsychosocial/ecological perspective, reviewing constructs across many levels in an approach often referred to as ‘from genes to groups’, ‘from neurons to neighborhoods’, or ‘from cells to culture’. It is testimony to the authors' erudition and vast knowledge. Moreover, it is engagingly written, making it an excellent research and teaching resource. Researchers and students of developmental psychology and psychopathology have gained a very valuable contribution.

Scholarly reviews are judged not only on their comprehensiveness, completeness, thoroughness, and up‐to‐date and integrative quality of the research discussed – the latter goal certainly accomplished by Hyde and colleagues – but also on their heuristic potential. Does the review inspire new questions and catalyze new research avenues that may extend and complement its scope? Can it become a springboard for new compelling questions? Can it forge new bridges and new rapprochements with other perspectives? Does it invite new ideas?

In this case, the answer is certainly affirmative. The review accomplishes much more than providing an excellent and up‐to‐date summary of what we know: It also fuels reflection and inspires intriguing new questions and directions of research, opening new exciting paths forward that might complement and further extend the rich tapestry so aptly woven by the authors. I would like to outline two new potential sets of ideas.

## Expanding the focus on early relational experience and parents' and children's emerging representations

Hyde et al.'s (2025) key focus is on origins of antisocial behaviors at preschool age. Although they do provide a very good review of several risk factors in infancy, for example certain temperament traits, and even certain prenatal characteristics, their emphasis appears centered on the maladaptive processes originating in the context of parent–child discipline and control. They describe very well and in depth the processes that give rise to the unfolding and entrenching coercive, adversarial dynamics. This view is consistent with research on coercive family systems, robustly supported by the social‐learning perspective; the account is clear, articulated very well, and grounded in the literature.

Of note, parental efforts to control and discipline the child and the child's responses, including defiance and other forms of noncompliance, typically emerge in the second year and rapidly increase thereafter. Although the authors' depiction of the coercive dynamics evolving in some parent–child dyads is, again, very accurate, I would argue that it underestimates the importance of the very critical first 2 years – before control and discipline fully ‘come online’.

Those very early years are the key time when the foundations of the parent–child relationship are being established in countless multifaceted daily interactions that encompass routines, play, meals, and various forms of care. In those contexts, infants express joy, fear, anger, sadness, or distress; they explore their environment, and they engage in social exchanges with the parents. As highlighted by attachment theory and research, qualities of parental response to a broad range of children's emotional and social cues are central for building the early relationship.

In secure relationships, children come to eagerly reciprocate parental responsiveness by also being responsive and cooperative toward the parents. This sets up the stage for the time when control and discipline do become salient – secure infants are likely to engage in willing compliance, cooperating with the parent. In insecure relationships, however, the developmental stage is set for the adversarial, coercive cycles, so aptly described by Hyde et al. (2025). Research on insecure attachment and future conduct problems has produced strong evidence that early insecurity is linked to future antisocial trajectories.

Research has also supported powerful moderating effects of (in)security. The legacy of early secure or insecure attachment, formed before the onset of parental control, can substantially influence the course of the bidirectional parent–child coercive, adversarial dynamics. In secure relationships, difficult children do not necessarily elicit harsh parenting, and harsh parenting does not necessarily have detrimental effects leading to conduct problems. But in insecure relationships, adversarial dynamics can be amplified, with parents and children primed to adopt confrontational, mutually resentful tactics.

Hyde et al. (2025) certainly do acknowledge the importance of parent–child attachment processes – both as a source of main effects and as a moderator of future socialization dynamics. But one may ask further questions about specific mechanisms through which early relational experience may impact children's conduct problems. I would like to propose an added emphasis on exploring the parent's and the child's representations of each other, emerging in their early forming relationship.

Interest in the representational aspects of the early parent–child relationship has been on the rise. Indeed, those are often seen as key mechanisms of the legacy of early relationships (Thompson, 2021). Attachment scholars believe that such representations, or internal working models (IWMs), are rooted in early relational experiences with the caregivers. Parents' IWMs, presumably resulting from their own early experiences of care, can be powerful guides of their parenting. Those representations, especially hostile attributions, negative relational schemas, resentment, and impoverished mentalization regarding the child lead to dysfunctional parenting, including rejection, harsh control, and ineffective or excessive discipline tactics in response to children's anger, distress, noncompliance, or defiance.

A complementary process may unfold on the part of the child. Children's IWMs of their parents, reflecting the history of care, originate very early in the relational experience. Children who, during the first year, come to view the parents as unresponsive and not trustworthy, after the onset of parental discipline, may be primed to perceive their control efforts as hostile, unfair, mean‐spirited, and arbitrary. They may resent and reject parents' influence attempts and socialization messages; and thus, the stage is set for a mutually adversarial cascade to unfold, ultimately leading to conduct problems (Kochanska, Boldt, & Goffin, 2019). Moreover, children's negative IWMs of their parents and of their early experience can generalize into broad hostile or defensive mindsets, strongly linked to antisocial conduct (Dodge, 2024).

Exploring the role of parents’ and children's early relational experience and their forming representations would not contradict Hyde et al.'s (2025) model; rather, it would complement and enrich it by proposing early mechanisms that set the stage for maladaptive socialization cascades leading to conduct problems. The authors do allude to this issue briefly, but further expansion would likely be an exciting research direction.

## Complementing the emphasis on adverse developmental factors with a focus on powerful positive socialization forces as buffering risks for antisocial behavior

Discussing the pathways to conduct problems, Hyde et al. (2025) quite naturally focus on the predominant presence of adverse, maladaptive, and dysfunctional processes. They depict those processes at all levels – the child, the caregiver, the dyad, and the broader ecology – see, for example, the excellent Figures 1 and 4, illustrating biopsychosocial cascades of unfolding risks across levels of analysis and timescales, respectively. Although they do mention on several occasions the role of positive socialization processes (e.g. warmth, emotion socialization, positive parenting and discipline strategies, security in the early relationship), they do so mostly to illustrate that those factors are absent or diminished in families in which children are at risk for conduct problems. This account is accurate, and it can be traced historically to Patterson's research on coercive family systems. Hyde et al.'s (2025) arguments are coherent, well stated, and robustly supported by data.

But it is theoretically possible to advance another conceptualization, one that would complement – without contradicting – the emphasis on negative socialization processes by highlighting a powerful role of positive factors. Such a new lens would align with the growing emphasis on positive forces in socialization and understanding that such forces are best seen not simply as markers of absent or diminished levels of the negative factors, but as alternative mechanisms that can be leveraged to support children's adjustment. Influenced by the positive psychology perspective, those forces are increasingly seen as potent contributors to children's positive development. Several dovetailing frameworks illustrate this perspective.

Historically, appreciation of positive socialization forces can be traced to Maccoby's pioneering ideas on the role of positive emotions (Maccoby & Martin, 1983), which continue to resonate today. Maccoby proposed a reformulation of the socialization process. She challenged the common views of socialization as a vaguely (or strongly) adversarial process, with the parent portrayed as pressuring the child toward a desired behavior and the child complying (or not). Instead, Maccoby posited that socialization can be a positive, mutually cooperative enterprise. Dovetailing with attachment theory, Maccoby proposed that a positive early parent–child relationship, imbued with shared positive emotions and an affectional bond, renders the child receptive to socialization and inaugurates a system of reciprocity, with both parent and child willingly collaborating toward shared goals. In such dyads, coercive, adversarial dynamics are unlikely to unfold.

One example inspired by that view is an alternative approach to ‘child effects’. It is known that child‐related factors, such as difficult, hard‐to‐manage temperament, contribute to conduct problems, as persuasively depicted by Hyde et al. (2025). However, a recently reformulated approach to child effects portrayed children as active agents in the positive sense: receptive, willing, even enthusiastic socialization partners, eagerly embracing parental goals and embarking on positive rather than antisocial trajectories. Such a view has been supported in multiple longitudinal studies (e.g., Kochanska, Kim, & Boldt, 2015).

Another example includes a rising focus on positive childhood experiences (e.g., Han, Dieujuste, Doom, & Narayan, 2023), conceptualized as independent from and not precluding the focus on adverse childhood experiences, with the latter largely dominating the literature on the etiology of children's conduct problems. Yet another example comes from the recent model of the role of interparental relationships. Research has amply documented the detrimental role of interparental conflict and hostility as spilling over to dysfunctional parenting and, in turn, leading to children's conduct problems, and that work is legitimately included in Hyde et al. (2025). But a new framework, Interparental Positivity Spillover Theory (Don, Simpson, Fredrickson, & Algoe, 2025), shifts the focus to positive interparental relationships, emphasizing the presence of elements such as various positive emotions, affection, laughter, humor, gratitude, or kindness. Those have positive effects on children's well‐being that are distinct from the effects of absent or low conflict and negativity. Positivity in interparental relationships promotes adaptive child outcomes and likely plays a significant protective role with regard to conduct problems. Further, those effects may be especially beneficial for children with certain traits (‘vantage sensitivity’; Pluess, 2024).

Of note, several successful prevention programs initiated during infancy and the toddler period already align with the perspective that emphasizes the importance of a positive parent–child relationship in infancy and early childhood (e.g., Circle of Security, Attachment and Biobehavioral Catch‐Up, Parent–Child Interaction Therapy, Family Check‐Up). Although diverse, they all share a focus on building strengths in early relational health.

Those theoretical and translational perspectives also dovetail with an emphasis on positive developmental outcomes such as skills, strengths, competencies, and well‐being, rather than simply absence or low levels of conduct problems (e.g., Pluess, 2024). Research programs that would complement the focus on adverse socialization trajectories and antisocial outcomes, so well synthesized by Hyde et al. (2025), by adding an emphasis on positive trajectories may greatly enrich our comprehensive understanding of children's adaptive and maladaptive development. Such research may also continue to have profound translational implications, as it would inform not only efforts to reduce risks but also interventions targeting specifically the positive socialization forces – expanding the prevention programs identified above and those yet to be developed.

In sum, Hyde et al. (2025) have given our field an invaluable, multifaceted, and stimulating contribution. We have gained an excellent and integrative review of the past and present of research on early conduct problems and an inspiring springboard for compelling new ideas. How exciting it is to imagine such future research enterprises.

## Ethical considerations

Ethical approval is not applicable to this Commentary Article. No data were used in this article.

**Key points**

- The review by Hyde at al. (2025), conducted through the lens of biopsychosocial/ecological perspective, is an impressive, cutting‐edge, impressive, comprehensive, scholarly, and up‐to‐date broad summary of the current state of developmental science related to origins of conduct problems.
- Perhaps even more importantly, it has a heuristic value, as it inspires new compelling questions, invites new ideas, and can potentially forge bridges with other perspectives and areas of research.
- Expanding the focus to relational experiences and parents' and children’s emerging representations in the first years of life can further elucidate very early origins of maladaptive cascades leading to conduct problems.
- Complementing the current emphasis on adverse developmental factors with research on positive socialization forces that can act as powerful buffers against risks for conduct problems can further enrich our understanding.

## Data Availability

Data sharing not applicable to this article as no datasets were generated or analyzed during the current study.
